# Racial/ethnic and family income related differences in dental radiographic utilization among a national representative sample of U.S. children and adolescents

**DOI:** 10.3389/froh.2025.1696551

**Published:** 2026-01-15

**Authors:** Vinodh Bhoopathi, Christine R. Wells, Sanjay Mallya

**Affiliations:** 1 Section of Public & Population Health, School of Dentistry, University of California at Los Angeles, Los Angeles, CA, United States; 2 Statistical Methods and Data Analytics, Office of Advanced Research Computing, University of California at Los Angeles, Los Angeles, CA, United States; 3 Section of Oral and Maxillofacial Radiology, School of Dentistry, University of California at Los Angeles, Los Angeles, CA, United States

**Keywords:** children, dental radiographs, dental visits, national survey of children's health, oral health disparities

## Abstract

**Background:**

For dental care professionals to accurately diagnose and prevent dental conditions, radiographic imaging is essential. However, the variation in dental radiograph use across different populations remains unclear. We examined national data on dental x-ray utilization and explored how racial/ethnic and family income related factors were associated among children and adolescents in the U.S.

**Methods:**

We conducted an observational cross-sectional analysis study of the National Survey of Children's Health (2016–2022) of children aged 1–17 years. The primary outcome variable indicates whether the child received a dental x-ray during the preventive dental visit in the past 12 months. We produced weighted estimates and fit survey-weighted adjusted logistic regression to estimate adjusted odds ratios (aORs) for dental x-ray receipt.

**Results:**

Out of 279,546 children, 56% received x-rays. Non-Hispanic Black children (aOR 0.55; 95% CI, 0.51–0.60), Hispanic children (aOR 0.90; 95% CI, 0.83–0.98), and children from other races/ethnicities (aOR 0.75; 95% CI, 0.70–0.80) were less likely to receive x-rays than non-Hispanic White children. Children from families with incomes below 200% FPL (aOR: 0.81, 95% CI: 0.75–0.88), and those earning between 200%–400% of the FPL (aOR: 0.92, 95% CI: 0.86–0.98) had a lower likelihood of receiving x-rays compared to families with incomes above 400% FPL.

**Conclusions:**

Dental radiography is commonly used during preventive visits, but its use in children depends on clinical need and age. In our study, we found significant disparities in dental radiograph utilization by race/ethnicity and family income.

## Introduction

Radiographic imaging is essential for diagnosing and planning dental care. These images provide unique information beyond clinical exams and supplement the patient's medical history and physical assessment. Dentists use radiographs to detect caries and evaluate tooth eruption, periodontal and endodontic disease, and other jaw pathoses (1). The need for radiographs and the type of radiographic examination are based on individual patient circumstances. Radiographs are often performed to assess particular symptoms or clinical signs. For example, an intraoral radiograph can evaluate a molar with deep caries and tenderness to percussion, while a panoramic radiograph is useful for examining a child with multiple unerupted teeth (1).

Radiographs may be used to screen for occult interproximal caries, even in the absence of symptoms or clinical disease (1). This helps detect early, untreated dental caries (2). Without radiographic information, diagnoses may be missed or incorrect, leading to inadequate treatment (3, 4). For example, the proximal tooth surfaces are not clinically accessible, and without bitewing radiography, interproximal caries lesions will not be detected, delaying preventive or restorative treatment (3, 4). To guide appropriate and timely radiographic imaging, the American Dental Association (ADA) and the American Academy of Oral and Maxillofacial Radiology (AAOMR) have developed criteria to select patients for imaging procedures (1, 5). These guidelines support the dentist in determining the necessity and type of radiographic exam needed based on the patient's history and clinical assessment. Following these guidelines is essential to maintain responsible, best-practice dental care.

Patients can maximize the benefits of diagnostic imaging in dental care by visiting a dental office and adhering to their dentist's guidance. However, diagnostic imaging is sparsely or underutilized. Studies across the broader medical field have revealed persistent disparities in diagnostic imaging across different sub-group of populations. For example, studies of pediatric emergency departments show that minority children are less likely to undergo radiographic imaging, such as head CT scans and abdominal ultrasounds, compared to their white counterparts, even after considering clinical presentation and illness severity (6, 7). Similar disparities occur in outpatient settings, influenced by factors like insurance coverage, language barriers, community characteristics, and the region where one lives, affecting access to advanced diagnostics (7). Systematic and scoping reviews in the medical literature also reveal disparities in the use of radiological services between children and adults (8–10). These differences in imaging access are worrisome because they can lead to delays in diagnosis, insufficient treatment, and poorer health outcomes in disadvantaged groups, increasing their vulnerability to higher disease morbidity.

Although much research has been conducted on disparities in healthcare imaging, few studies have addressed similar issues in dental care. A 2004 study of dentate adults revealed that Black Americans and those from low-income backgrounds were considerably less likely to undergo comprehensive dental imaging than their counterparts (11). Previous research has examined disparities in preventive dental care among children; however, studies on preventive dental x-ray utilization are scarce, let alone at the national level. Therefore, we analyze nationally representative data on U.S. children and adolescents aged 1–17 to evaluate the prevalence of dental x-ray use in the past year. Given that the medical literature indicates disparities in radiographic utilization attributable to factors such as race/ethnicity and family income, we investigate disparities in dental x-ray utilization among children and adolescents by race/ethnicity and family income.

## Methods

For this observational cross-sectional study, we used publicly available data from 2016 to 2022 from the National Survey of Children's Health (NSCH). This dataset is a nationally representative sample of noninstitutionalized children aged 1–17 in the United States (12–14). The overall analytical sample included data from 279,546 children and adolescents (referred to as “children” hereafter). We selected relevant variables from the topical questionnaire, which was completed by a parent or caregiver (referred to as “parents” hereafter) familiar with the child's health. We followed the Strengthening the Reporting of Observational Studies in Epidemiology (STROBE) guidelines in reporting our observational study.

### Main outcome variable

The parents were asked, “During the past 12 months, did this child see a dentist or other oral health care provider for any kind of dental or oral health care?”. Those who indicated their child had a dental visit in the past 12 months were further asked if their child received preventive dental care during that visit. Parents who answered affirmatively were prompted to respond if their child received preventive dental care services, with one of the options being “x-rays”.

### Main independent variables

We used two main independent variables. Race/ethnicity was categorized into: Hispanic, non-Hispanic White, non-Hispanic Black, and other. The primary language spoken at home was classified as English, Spanish, or other. The federal poverty level (FPL) or family income level was another categorical variable, categorized as less than 200% FPL, between 200% and 400% FPL, and over 400% FPL.

### Other covariates

Covariates were selected based on the existing medical (6–10) and dental (11) literature on radiographic utilization among children and adolescents, as well as on data availability in NSCH. The following covariates were used: age [1–5, 6–10, 11–13, and 14–17], sex [male or female], insurance coverage [public only, private only, both private and public, unspecified insurance type, and uninsured],.highest education level attained by a person living within the household [less than high school, high school or General Educational Development (GED), some college or technical school, and having a college degree or higher], and the geographic location where children lived [rural, urban, and suburban areas] (15). Two self-reported oral health complications were used. Parents were asked: “During the past 12 months, has this child had frequent or chronic difficulty with any of the following?” We included two options (toothaches and tooth decay) for our study.

### Statistical data management and analyses

We created a new variable to capture “oral health complications” by summing the positive responses to the question regarding difficulties experienced in the past year due to toothaches or tooth decay. This variable was divided into three categories: two oral health complications, one oral health complication, and no oral health complications. All analyses incorporated the survey elements provided by NSCH, including survey weights. First, we assessed the overall demographics of the children in our sample, using weighted estimates. We then examined the use of x-rays among different child subgroups as defined by the two primary independent variables (Race/ethnicity and family income), and other covariates. Finally, we constructed a logistic regression model to assess associations, using adjusted odds ratios (aOR), between the two main independent variables and x-ray use during dental visits in the past 12 months, adjusting for other covariates.

All the confounders we included *a priori* were selected based on their associations to radiographic utilization from existing medical and dental literature (6–11). We included both family income and insurance status because they influence access to dental care and diagnostic resources, impacting coverage limits, costs, provider participation, and radiographic services availability. They are also linked to oral disease risks and care-seeking behaviors, influencing the need for radiographs. These variables impact both socioeconomic factors and radiographic use. Missing data were handled via listwise deletion. Data management and analysis were performed using Stata 19.5 (Statacorp, College Station, TX) (16). A significance level of 0.05 was used in this analysis.

## Results

Approximately 56% reported that their child had received dental x-rays during a preventive dental visit within the past 12 months. Table 1 summarizes the characteristics of the overall analytical sample and the adjusted distribution of x-ray receipt. Non-Hispanic White children had the highest x-ray receipt rate (62%), while Non-Hispanic Black children had the lowest (46%). Only 48.5% of children from families earning less than 200% of the FPL received x-rays, compared to 61% from families earning more than 400% of the FPL (Table 1). The receipt of x-rays varied significantly by age, with the highest percentage occurring in children aged 11–13 years (65%) and the lowest in those aged 1–5 years (30%). Sex differences were minimal, as 56% of males and 56.5% of females received x-rays. Children from English-speaking households showed higher x-ray receipt rates (59%) compared to those from Spanish-speaking households (39%) and other language households (43%). Children with only private insurance were more likely to have received x-rays (62%) compared to those with only public insurance (49%), those with both public and private insurance (51%), or those without insurance (45%). The education level of the household also played a crucial role: children from families with a college degree or higher had a higher rate of x-ray receipt (61%) than those whose parents had less than a high school education (42%). Regional differences were noted, with children in suburban areas showing the highest x-ray receipt (58.5%), followed by urban (53%) and rural areas (53%). Children reporting frequent or chronic difficulties due to decayed teeth (66%) or toothaches (63%) had higher x-ray receipts than those without such complications (Table 1).

**Table 1:** Characteristics of the analytical sample stratified by receipt of x-rays during the dental visit in the past 12 months.

Characteristics	Overall (*n* = 269,767)	Receipt of x-rays during preventive dental visit in the past 12 months	

	Percentage (%)	Yes (%)	No (%)
Age			
1–5	28.4	29.9	70.1
6–10	29.3	62.3	37.7
11–13	18.3	65.2	34.8
14–17	24.0	63.2	36.8
Sex			
Male	51.1	56.0	44.0
Female	48.9	56.5	43.5
Race/ethnicity			
Hispanic	25.6	50.3	49.7
Non-Hispanic White	50.1	62.2	37.8
Non-Hispanic Black	13.2	46.3	53.7
Other	11.1	53.6	46.4
Language spoken in house			
English	85.2	58.8	41.2
Spanish	10.1	39.2	60.8
Other	4.7	43.0	57.0
Type of insurance coverage			
Public only	30.4	48.8	51.2
Private only	58.1	61.8	38.2
Private and Public	4.6	50.8	49.2
Insurance type not specified	0.2	39.0	61.0
Not insured	6.7	44.6	55.4
Highest Education level in household			
Less than high school education	9.3	41.6	58.4
High school or GED	19.4	49.2	50.8
Some college or technical school	21.3	55.4	44.6
College degree or higher	50.0	61.4	38.6
Federal poverty level			
Less than 200%	34.6	48.5	51.5
200 to 400%	24.1	58.1	41.9
More than 400%	41.3	61.0	39.0
Geographic Location			
Rural	11.5	53.2	46.8
Suburban	55.5	58.5	41.5
Urban	33.0	53.4	46.6
Frequent or chronic difficulty in last 12 months due to decayed teeth			
Yes	11.9	66.2	33.8
No	88.1	54.8	45.2
Frequent or chronic difficulty in last 12 months due to tooth aches			
Yes	4.2	62.8	37.2
No	95.8	56.0	44.0
Received x-rays during the dental visit in the past 12 months			
Yes	56.3		
No	43.7		

**Figure 1:**
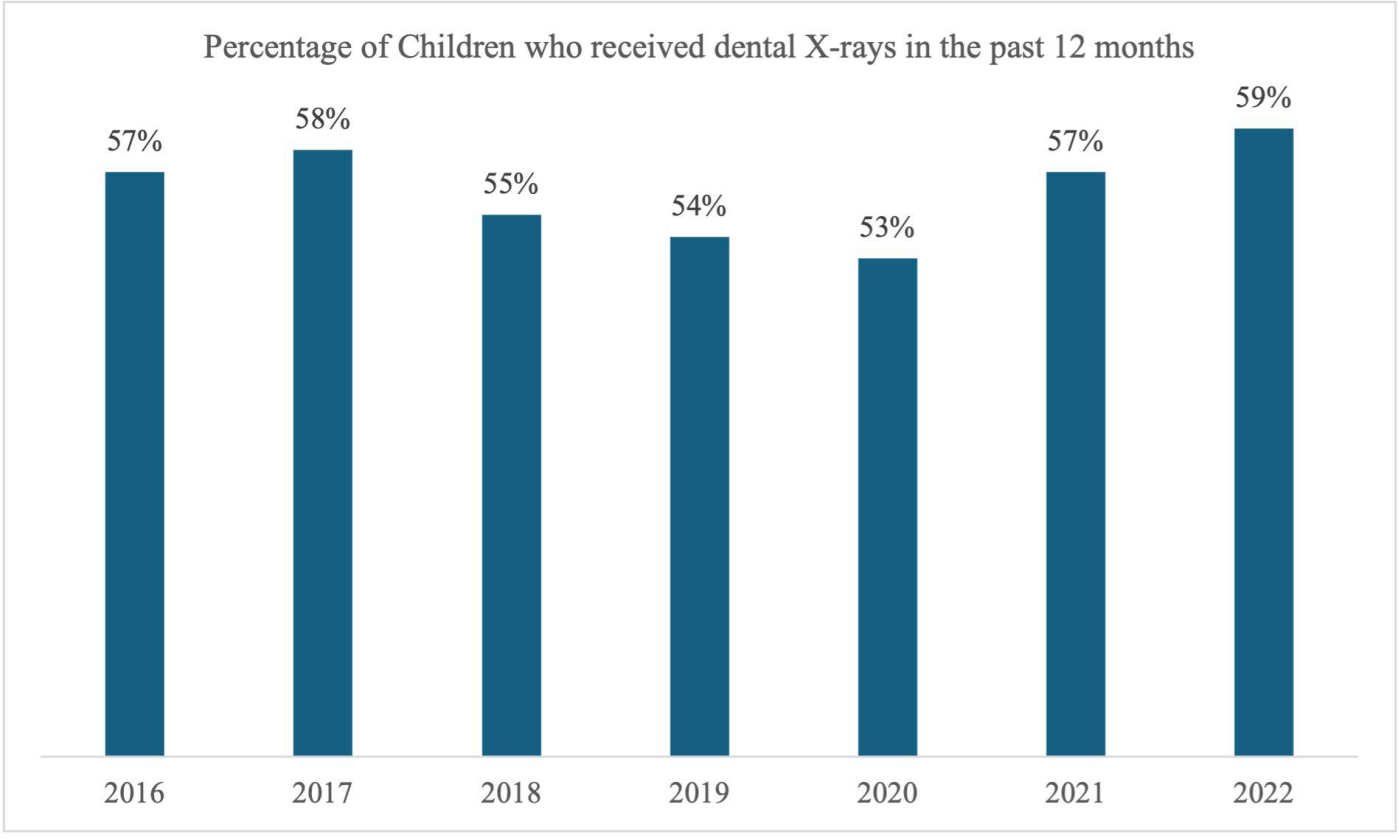
Percentage of children receiving dental x-rays in the past 12 months between 2016 and 2022.

Figure 1 illustrates how dental x-ray usage among children and adolescents has changed each year from 2016 to 2022. The percentage of children and adolescents aged 1–17 years who received dental x-rays during preventive visits varied from 53% in 2020 to 59% in 2022. Table 2 shows the yearly descriptive statistics from 2016 to 2022 for the outcome variable, key independent variables, and additional covariates, along with the sample size.

**Table 2:** Descriptive statistics of children aged 1 through 17 years by year of the survey.

Characteristics	2016 (*n* = 50,122)	2017 (*n* = 21,599)	2018 (*n* = 30,530)	2019 (*n* = 29,433)	2020 (*n* = 42,777)	2021 (*n* = 50,892)	2022 (*n* = 54,103)
Age (%)							
1–5	28.6	28.6	28.5	28.6	28.5	28.5	27.4
6–10	29.8	29.7	29.6	29.1	28.9	28.9	29.0
11–13	17.6	17.8	18.0	18.4	18.6	18.6	18.7
14–17	23.9	23.8	23.9	23.9	24.0	24.0	24.9
Sex (%)							
Male	51.1	51.1	51.1	51.1	51.1	51.1	51.2
Female	48.9	48.9	48.9	48.9	48.9	48.9	48.9
Race/ethnicity (%)							
Hispanic	24.6	24.9	25.4	25.8	25.8	25.9	27.0
Non-Hispanic White	51.9	50.8	50.4	50.1	49.9	49.8	47.8
Non-Hispanic Black	12.8	13.7	13.4	13.3	13.2	13.4	12.6
Other	10.7	10.6	10.8	10.9	11.0	10.8	12.6
Language spoken in house (%)							
English	86.0	85.4	85.0	86.1	85.0	84.1	85.0
Spanish	9.6	9.5	10.5	9.3	10.5	11.1	10.1
Other	4.4	5.1	4.5	4.6	4.5	4.8	4.9
Type of insurance coverage (%)							
Public only	31.2	30.9	31.0	29.2	29.6	30.5	30.4
Private only	56.8	57.8	58.4	59.3	58.2	58.1	57.9
Private and Public	4.3	5.0	4.0	4.8	4.9	4.0	5.0
Insurance type not specified	1.7	0.0	0.0	0.0	0.0	0.0	0.0
Not insured	6.0	6.3	6.6	6.7	7.3	7.4	6.7
Highest Education level in household (%)							
Less than high school education	9.4	8.7	9.9	9.4	9.7	9.7	8.7
High school or GED	19.5	20.0	19.1	18.8	19.5	19.8	18.8
Some college or technical school	22.6	22.3	21.9	21.8	20.6	19.6	20.1
College degree or higher	48.5	49.0	49.1	50.0	50.2	50.9	52.4
Federal poverty level (%)							
Less than 200%	44.1	42.7	41.3	40.3	39.7	39.6	38.3
200 to 400%	26.7	26.6	27.3	28.7	29.4	28.5	28.6
More than 400%	29.2	30.7	31.4	31.0	30.9	31.9	33.1
Geographic Location (%)							
Rural	11.6	10.1	11.8	11.3	11.9	11.9	11.9
Suburban	55.3	55.1	55.4	56.9	55.0	54.9	55.6
Urban	33.1	34.9	32.8	31.8	33.1	33.2	32.4
Oral Health Complications (Tooth decay and Tooth aches)							
One complication	10.6	11.1	10.7	10.8	10.8	10.7	10.8
Two complications	2.2	2.2	2.8	2.3	2.8	2.9	2.6
No complications	87.2	86.7	86.5	86.9	86.4	86.4	86.6
Received x-rays during the dental visit in the past 12 months							
Yes	56.7	58.4	55.0	54.1	53.0	57.1	59.4
No	43.3	41.6	45.0	45.9	47.0	42.9	40.6

In the logistic regression analysis (see Table 3), after adjusting for covariates, race/ethnicity and family income were independently associated with dental x-ray utilization. Non-Hispanic Black children were 45% less likely to receive x-rays than Non-Hispanic White children (aOR = 0.55, 95% CI: 0.51–0.60). Similarly, Hispanic children (aOR = 0.90, 95% CI: 0.83–0.98) and children from other racial backgrounds (aOR = 0.75, 95% CI: 0.70–0.80) also had lower odds of getting x-rays compared to their Non-Hispanic White peers. Family income levels influenced the receipt of x-rays. Children from families with incomes below 200% FPL (aOR: 0.81, 95% CI: 0.75–0.88), and those earning between 200%–400% of the FPL (aOR: 0.92, 95% CI: 0.86–0.98) had a lower likelihood of receiving x-rays compared to families with incomes above 400% FPL.

**Table 3:** Multivariable logistic regression model of receipt of x-rays during preventive dental visits in the past 12 months.

Characteristics	Receipt of x-rays during the dental visit in the past 12 months	
	AOR (95% CI)	*p*-value
Age		**<0** **.** **0001**
6–10	3.92 (3.65–4.20)	**<0** **.** **0001**
11–13	4.82 (4.46–5.22)	**<0** **.** **0001**
14–17	4.36 (4.06–4.68)	**<0** **.** **0001**
1–5	(Reference)	
Sex		
Female	1.02 (0.98–1.07)	0.31
Male	(Reference)	
Race/ethnicity		**<0** **.** **0000**
Hispanic	0.90 (0.83–0.98)	**0** **.** **01**
Other	0.75 (0.70–0.80)	**<0** **.** **0001**
Non-Hispanic Black	0.55 (0.51–0.60)	**<0** **.** **0001**
Non-Hispanic White	(Reference)	
Language spoken in house		**<0** **.** **0000**
Spanish	0.54 (0.47–0.63)	**<0** **.** **0001**
Other	0.62 (0.54–0.71)	**<0** **.** **0001**
English	(Reference)	
Type of insurance coverage		**<0** **.** **0000**
Public only	0.90 (0.83–0.97)	**0** **.** **007**
Private and Public	0.82 (0.72–0.93)	**0** **.** **001**
Insurance type not specified	0.59 (0.31–1.13)	0.11
Not insured	0.60 (0.52–0.69)	**<0** **.** **0001**
Private only	(Reference)	
Highest Education level in household		**<0** **.** **0000**
High school or GED	1.14 (0.98–1.34)	0.09
Some college or technical school	1.35 (1.16–1.57)	**<0** **.** **0001**
College degree or higher	1.59 (1.36–1.85)	**<0** **.** **0001**
Less than high school education	(Reference)	
Federal poverty level		**<0** **.** **000**
Less than 200%	0.81 (0.75–0.88)	**<0** **.** **0001**
200 to 400%	0.92 (0.86–0.98)	**0** **.** **009**
More than 400%	(Reference)	
Geographic Location		**0** **.** **0000**
Suburban	1.01 (0.95–1.07)	0.74
Rural	0.79 (0.74–0.85)	**<0** **.** **0001**
Urban	(Reference)	
Oral Health Complications (Tooth decay and Tooth aches)		**<0** **.** **000**
One complication	1.86 (1.69–2.04)	**<0** **.** **0001**
Two complications	2.07 (1.73 -2.48)	**<0** **.** **0001**
No complications	(Reference)	

NOTE: *P*-value of omnibus tests are given on the row with the variable name. All omnibus tests were statistically significant (except sex). Bold value means statistically significant.

## Discussion

Our research reveals significant racial/ethnic and socioeconomic disparities in the use of dental x-rays among U.S. children and adolescents, These findings expand upon existing disparities observed in other types of medical imaging studies.

In our study, Non-Hispanic Black children exhibited the lowest rates of x-ray utilization, a pattern observed in numerous clinical environments where minority groups often face barriers to diagnostic services (9). Prior research has identified racial and ethnic disparities in preventive dental care (17, 18). Systemic biases, socioeconomic challenges, and limited access to quality healthcare in minority communities likely drive these disparities. Although not specifically documented in dental studies, a few research efforts have also noted instances of overuse or inappropriate radiographic examinations among minority populations compared to white groups (19, 20). Children from higher-income families had higher rates of receiving dental x-rays, suggesting that financial resources play a key role in accessing dental diagnostic services. This indicates that the uninsured and those with public insurance face significant barriers to accessing timely and comprehensive dental care (8).

In addition to the above findings, we highlight associations between other covariates and the receipt of dental x-rays. Children and adolescents aged 6–17 are more likely to undergo dental radiographs compared to those aged 1–5 years. This suggests that dental practitioners adhere to guidelines recommending radiographs commencing at age 6, when permanent dentition begins to erupt, in order to evaluate the mixed dentition and screen for interproximal caries (1). As individuals mature, the incidence of dental caries and oral health issues increases, thereby augmenting the necessity for diagnostic x-rays. A study involving individuals aged 1–11 demonstrated that the utilization of x-rays escalates with age, particularly during adolescence, attributable to the requirement for comprehensive and preventive dental care (21).. Indicators such as toothaches and cavities strongly predict the need for dental radiographs, indicating that imaging is used appropriately. Our findings align with prior studies linking radiographic prescriptions to insurance claims, underscoring the need for clinical use (22). Our research shows children with private insurance are more likely to get dental x-rays than those with public or no insurance, highlighting insurance's role in access. Prior studies confirm that private insurance users utilize more preventive and diagnostic healthcare services, including dental imaging (11).

A large sample size, pooling multi-year NSCH data, and adjustment for confounders are all strengths of our study. We also acknowledge the study's limitations.

The data were parent-reported radiograph receipt of their children; therefore, misclassification or recall bias is possible. NSCH does not collect clinical data, which could have validated some parent-reported clinical complications used in this study.
We used cross-sectional data; therefore, no causal inference was made, although that was not the intent of our analyses.
The survey did not distinguish among intraoral, panoramic, or cone-beam computed tomography (CBCT) imaging, although these data likely represent 2-dimensional imaging. The survey instrument lists “x-rays” as a single category of procedures. The individual imaging examinations (intraoral, panoramic and CBCT) are performed for various diagnostic indications. Intraoral and panoramic radiography are basic imaging examinations that provide information about the teeth and jaws. CBCT examinations are made for advanced pathoses and more advanced treatment applications. The lack of specifics of the examination type limits our ability to make precise determinations on the impact of missed radiography on oral health
Our data did not include the provider's specialty. Previous studies (22, 23) have found that pediatric dentists are more likely than general dentists to prescribe radiographs in younger children.

In summary, across this nationally representative NSCH sample, just over half of children received dental x-rays at a preventive visit. However, utilization varied significantly by race/ethnicity and income, with non-Hispanic Black, Hispanic, and children of other races consistently showing lower adjusted odds compared to non-Hispanic White children and those from lower-income families.

These gaps suggest the need to ensure ADA/AAOMR radiographic selection criteria are applied consistently across racial/ethnic groups and income strata, using standardized indication documentation and clinic-level quality checks (e.g., auditing radiograph use by race/ethnicity and FPL) so that children with comparable risk/need are equally likely to receive indicated imaging.

Because lower income (<200% FPL) is linked to lower receipt, while higher income groups have greater odds, the findings support policies that reduce financial and structural barriers to diagnostic imaging in low-income communities (coverage design that limits out-of-pocket costs for indicated imaging and investments that expand diagnostic capacity in clinics serving low-income and racially/ethnically diverse populations).

As mentioned above, systemic biases such as provider bias (often implicit) may contribute to disparities in dental x-ray utilization related to race/ethnicity and income. These disparities could stem from differences in clinical decision-making, communication, or assumptions about follow-through and affordability. Training and educating the dental workforce on implicit bias and cultural competency is essential to reduce unintentional bias and, consequently, disparities.

## Data Availability

Publicly available datasets were analyzed in this study. This data can be found here: https://www.childhealthdata.org/learn-about-the-nsch/NSCH.

## References

[1] American Dental Association Council on Scientific Affairs and U.S. Food and Drug Administration. Dental Radiographic Examinations: Recommendations for Patient Selection and Limiting Radiation Exposure. Chicago, IL: American Dental Association (2012). Available online at: https://www.fda.gov/media/84818/download (Accessed August 20, 2025).

[2] Chou R Selph SS Bougatsos C Nix C Ahmed A Griffin J Screening, Referral, Behavioral Counseling, and Preventive Interventions for Oral Health in Adults: A Systematic Review for the U.S. Preventive Services Task Force. Rockville (MD): Agency for Healthcare Research and Quality (US) (2023). (Evidence Synthesis, No. 233.) Available online at: https://www.ncbi.nlm.nih.gov/books/NBK597289/ (Accessed August 10, 2025). 37972224

[3] Schwendicke F Göstemeyer G. Conventional bitewing radiographs. In: Ferreira Zandona A Longbottom C, editors. Detection and Assessment of Dental Caries: A Clinical Guide. Cham: Springer International Publishing (2019). p. 109–17.

[4] Dove SB. Radiographic diagnosis of dental caries. J Dent Educ. (2001) 65(10):985–90. 10.1002/j.0022-0337.2001.65.10.tb03474.x 11700001

[5] Tyndall DA Price JB Tetradis S Ganz SD Hildebolt C Scarfe WC. Position statement of the American academy of oral and maxillofacial radiology on selection criteria for the use of radiology in dental implantology with emphasis on cone beam computed tomography. Oral Surg Oral Med Oral Pathol Oral Radiol. (2012) 113(6):817–26. 10.1016/j.oooo.2012.03.005 22668710

[6] Marin JR Rodean J Hall M Alpern ER Aronson PL Chaudhari PP Racial and ethnic differences in emergency department diagnostic imaging at US children’s hospitals, 2016–2019. JAMA Netw Open. (2021) 4(1):e2033710. 10.1001/jamanetworkopen.2020.33710 33512517 PMC7846940

[7] Ross AB Kalia V Chan BY Li G. The influence of patient race on the use of diagnostic imaging in United States emergency departments: data from the national hospital ambulatory medical care survey. BMC Health Serv Res. (2020) 20:840. 10.1186/s12913-020-05698-1 32894129 PMC7487740

[8] Betancourt JR Tan-McGrory A Flores E López D. Racial and ethnic disparities in radiology: a call to action. J Am Coll Radiol. (2019) 16(4 Pt B):547–53. 10.1016/j.jacr.2018.12.024 30947886

[9] Colwell RL Narayan AK Ross AB. Patient race or ethnicity and the use of diagnostic imaging: a systematic review. J Am Coll Radiol. (2022) 19(4):521–8. 10.1016/j.jacr.2022.01.008 35216945

[10] DeBenedectis CM Spalluto LB Americo L Bishop C Mian A Sarkany D Health care disparities in radiology-A review of the current literature. J Am Coll Radiol. (2022) 19(1 Pt B):101–11. 10.1016/j.jacr.2021.08.024 35033297

[11] Gilbert GH Coke JM Weems RA Shelton BJ. Patient characteristics associated with receipt of dental radiographic procedures during a 48-month population-based study of dentate adults. Oral Surg Oral Med Oral Pathol Oral Radiol Endod. (2004) 97(5):642–51. 10.1016/j.tripleo.2003.10.016 15153879

[12] US Census Bureau. Methodology and Data User FAQs. Available online at: https://www.census.gov/programs-surveys/nsch/technical-documentation/methodology.2016.html#list-tab-184951139 (Accessed December 10, 2025).

[13] US Census Bureau. Methodology and Data User FAQs. Available online at: https://www.census.gov/programs-surveys/nsch/technical-documentation/methodology.2019.html#list-tab-184951139 (Accessed December 10, 2025).

[14] US Census Bureau. Methodology and Data User FAQs. Available online at: https://www.census.gov/programs-surveys/nsch/technical-documentation/methodology.2022.html#list-tab-184951139 (Accessed December 10, 2025).

[15] Mantey DS Omega-Njemnobi O Hunt ET Lanza K Cristol B Kelder SH. Home smoke-free policies as children age: urban, rural, and suburban differences. Nicotine Tob Res. (2022) 24(12):1985–93. 10.1093/ntr/ntac186 35901848 PMC9653085

[16] StataCorp. StataNow Statistical Software: Release 19.5. College Station, TX: StataCorp LLC (2025).

[17] Luo H Moss ME Wright W Webb M Pardi V Lazorick S. Racial/ethnic disparities in preventive dental services use and dental caries among children. J Public Health Dent. (2023) 83(2):161–8. 10.1111/jphd.12563 36883255 PMC10258156

[18] Robison V Wei L Hsia J. Racial/ethnic disparities among US children and adolescents in use of dental care. Prev Chronic Dis. (2020) 17:E71. 10.5888/pcd17.190352 32730202 PMC7417021

[19] Falchook AD Hendrix LH Chen RC. Guideline-discordant use of imaging during work-up of newly diagnosed prostate cancer. J Oncol Pract. (2015) 11:e239–46. 10.1200/JOP.2014.001818 25670199

[20] Wood JN Hall M Schilling S Keren R Mitra N Rubin DM. Disparities in the evaluation and diagnosis of abuse among infants with traumatic brain injury. Pediatrics. (2010) 126:408–14. 10.1542/peds.2010-0031 20713477

[21] Mahabee-Gittens EM Smith HA Merianos AL. Disparities in dental health issues and oral health care visits in US children with tobacco smoke exposure. J Am Dent Assoc. (2022) 153(4):319–29. 10.1016/j.adaj.2021.09.002 35078590 PMC8969190

[22] Fontana M Yepes JF Eckert GJ Hale KJ Benavides E. Patterns of radiograph use in a population of commercially insured children. J Am Dent Assoc. (2022) 153(5):405–13. 10.1016/j.adaj.2021.09.014 35125167

[23] Menaker NH Yepes JF Vinson LA Jones JE Downey T Tang Q Prescription of bite-wing and panoramic radiographs in pediatric dental patients: an assessment of current trends and provider compliance. J Am Dent Assoc. (2022) 153(1):23–30. 10.1016/j.adaj.2021.07.001 34654530

